# A global molecular phylogeny yields insights into the dispersal and invasion history of *Junonia*, a butterfly genus with remarkable dispersal abilities

**DOI:** 10.1098/rspb.2021.2801

**Published:** 2022-06-08

**Authors:** Melanie M. L. Lalonde, Jeffrey M. Marcus

**Affiliations:** Department of Biological Sciences, University of Manitoba, Winnipeg, Manitoba, Canada

**Keywords:** *Junonia*, mitogenomics, Junoniini, phylogenomics, reticulate evolution, evolutionary radiation

## Abstract

The nymphalid butterfly genus *Junonia* has remarkable dispersal abilities. Occurring on every continent except Europe and Antarctica, *Junonia* are often among the only butterflies on remote oceanic islands. The biogeography of *Junonia* has been controversial, plagued by taxonomic disputes, small phylogenetic datasets, incomplete taxon sampling, and shared interspecific mitochondrial haplotypes. *Junonia* originated in Africa but its route into the New World remains unknown. Presented here is, to our knowledge, the most comprehensive *Junonia* phylogeny to date, using full mitogenomes and nuclear ribosomal RNA repeats from 40 of 47 described species. *Junonia* is monophyletic and the genus *Salamis* is its probable sister clade. Genetic exchange between Indo-Pacific *Junonia villida* and New World *Junonia vestina* is evident, suggesting a trans-Pacific route into the New World. However, in both phylogenies, the sister clades to most New World *Junonia* contain both African and Asian species. Multiple trans-Atlantic or trans-Pacificinvasions could have contributed to New World diversification. Hybridization and lateral transfer of mitogenomes, already well-documented in New World *Junonia*, also occurs in at least two Old World lineages (*Junonia orithya*/*Junonia hierta* and *Junonia iphita/Junonia hedonia*). Variation associated with reticulate evolution creates challenges for phylogenetic reconstruction, but also may have contributed to patterns of speciation and diversification in this genus.

## 1. Introduction

The balance between immigration, extinction and diversification determines species richness in geographical localities [1]. Most immigrants fail to establish populations, and the majority that colonize successfully undergo little or no diversification [2]. Only rare immigrant lineages show high diversification rates and adaptive radiation [2]. Understanding factors that make immigrant lineages successful in new habitats is important to the disciplines of biogeography, invasion biology, evolution and conservation. The butterfly genus *Junonia* has extraordinary abilities for dispersal and diversification. *Junonia* originated 15–27 million years ago (Ma) [3] and are often among the few butterfly species present on remote oceanic islands [4,5], suggesting that they can survive crossing thousands of kilometers of open water to colonize and diversify in new habitats. Some Old World *Junonia* (28 extant species) appear to have crossed an ocean basin 2–4 Ma to establish a radiation of 18 New World species [3]. This unparalleled ability to disperse may have fomented diversification in *Junonia*, but the lack of effective geographical barriers to gene flow also may have stymied previous attempts to resolve its phylogenetic and biogeographic history.

**Figure 1.**
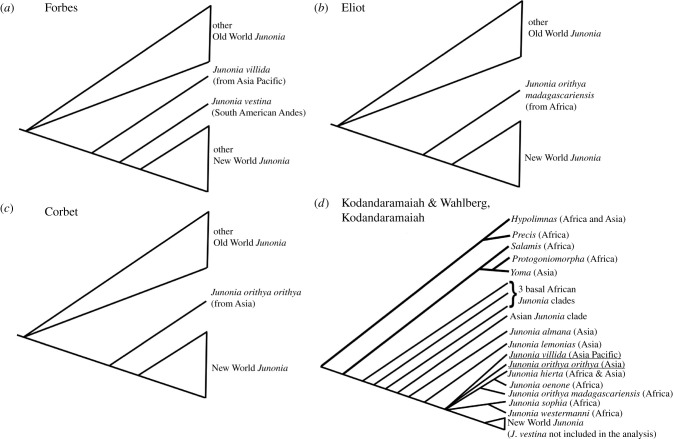
Hypotheses for the phylogeny of *Junonia* with proposed sister groups to the New World *Junonia*: (*a*) *Junonia villida* by Forbes [14,15], (*b*) African *Junonia* *orithya madagascariensis* by Eliot [12,13] and (*c*) Asian *Junonia orithya orithya* by Corbet [11] based on morphology; (*d*) presents the molecular phylogeny of Kodandaramaiah & Wahlberg [3] and Kodandaramaiah [9] as interpreted by McCullagh [10].

*Junonia* has a convoluted taxonomic history, especially for New World species. This is attributable to conflation of the generic names *Precis* (restricted to a related African clade) and *Junonia* (distributed nearly worldwide) by some authors, misidentifications owing to intraspecific seasonal and geographical variation, loss or absence of type specimens, and the failure of many authors to reference taxonomic authorities used for specimen identification [6–8]. Consequently, creating a robust phylogeny for the genus *Junonia* has been difficult. Several morphology-based phylogenetic hypotheses are mutually contradictory (figure 1*a–c*; [11–15]). They also differ from molecular phylogenies based on small mitochondrial and nuclear sequence datasets (*COI*, *wingless* and *EF1-alpha*) (figure 1*d*; [3,9,16–19]). Finally, New World mitochondrial DNA haplotypes are often shared among all sympatric species in any given locality, resulting in identical heterospecific mitochondrial genome sequences [6,17–20].

Morphology-based taxonomic assignments have become easier owing to recent clarification of New World species taxonomy [6,7,17,20–23], but remain challenging for reasons described above, and owing to poorly described, undescribed and cryptic *Junonia* species [20,23]. In general, *Junonia* phylogenetic studies have either contained very few (≤3) New World species [9,24,25], or (≤2) Old World species [19,23]. Further, some phylogenetic studies of New World taxa have included GenBank sequences labelled as either *Junonia evarete* or *Junonia genoveva*, but in the absence of museum vouchers it is unclear if the source-specimens were correctly identified or if they belong to one of six other potential *Junonia* species [6,16–19]. The use of DNA barcoding [3,17–19] and later, full mitochondrial genome sequencing [5,26–28], confirmed that *Junonia* is a monophyletic clade. Although still missing species, these studies improved phylogenetic resolution over earlier studies.

The invasion of the New World by *Junonia* has been the subject of decades of speculation. Based on molecular phylogenetic and biogeographic analyses, the consensus is that *Junonia* originated in Africa [3,19], but which lineages invaded the New World remains unanswered. Four taxa have been suggested as sister to the New World *Junonia*: *Junonia villida* (Indo-Pacific) [15], *Junonia orithya madagascariensis* (Africa) [12,13], *Junonia orithya orithya* (Asia) [11] and *Junonia lemonias* (Asia) [5,10]. Also unknown is whether the occupation of the New World by *Junonia* was a unique event [3,8,12–15,19] or caused by multiple invasions involving one or more Old World species [10,18]. Recent full mitochondrial genome sequence data suggests a trans-Pacific route and that the same lineage (*J. villida*) crossed the Pacific more than once to reach the New World [5,10].

Individual Old World *Junonia* species have been considered to be monophyletic based on morphology and molecular characters [3,9,24,29]. By contrast, New World *Junonia* species do not form monophyletic clades based on most molecular studies [5,6,10,17–20,23,26]. Instead, all New World *Junonia* species tend to share mitochondrial haplotypes and sympatric populations of *Junonia* species typically show the same mitochondrial haplotype group frequencies [18,30,31]. Many New World *Junonia* species have the capacity to interbreed, so hybridization and mitochondrial introgression events are possible [20,23,32,33]. Consequently, mitochondrial gene-based phylogenies are not useful for New World species delimitation [3,5,6,9,17–20,23], but if species determination is first done using other characteristics, the study of mitochondrial haplotype distributions permits characterization of biogeographic patterns of genetic variation [18,20,30].

### (a) DNA barcodes, short fragment nuclear DNA and haplotype groups

Three primary mitochondrial haplotype groups are shared among New World *Junonia* species [3,6,9,16–20,23]. This pattern was first discussed by Pfeiler *et al*. [19] based on the 658 base pair *cytochrome oxidase I* DNA barcoding fragment used to delimit many animal species [34,35]. Pfeiler *et al*. [19] suggested that shared mitochondrial haplotypes may be owing to the recent invasion of *Junonia* into the New World (approx. 2–4 Ma) [3], such that lineage sorting is incomplete and hybridization between species is ongoing. Haplotype group A is common in South American and Caribbean *Junonia*, while haplotype group B is common in North and Central America*,* and these two monophyletic groups diverged approximately 2.2 Ma [19]. Pfeiler *et al*. [19] found that *Junonia vestina*, a high-elevation South American species, had a distinct sequence (called A_1_) within haplotype group A and considered all other species to carry subgroup A_2_. Based on outgroup selection, Pfeiler *et al*. [19] speculated that the sister taxon to New World *Junonia* may have been an African lineage related to *J. orithya* or *Junonia westermanni*, although this was based on very limited sampling of Old World species.

Later, Gemmell & Marcus [18] mapped the New World distribution patterns of haplotype groups and confirmed that Californian *Junonia grisea* specimens carried a distinct variant (B^CA^) of haplotype group B. Haplotype B^CA^ also occurs at a low frequency in four other American southwest *Junonia* species [20]. Gemmell & Marcus [18] suggested that mitochondrial DNA sequences from Indo-Pacific *J. villida* are more similar to the New World *Junonia* than sequences from *J. orithya*, consistent with some previous hypotheses based on morphology and geographical distributions [4,14,36]. They proposed two hypotheses for the origin of haplotype B: either it evolved from haplotype group A mitochondria within the New World, or that two separate New World invasions carried haplotype groups A and B, followed by hybridization between species descended from the two invasion events [18].

### (b) The use of full mitochondrial DNA genomes

Mitochondrial genome (mitogenome) sequences are a rich source of genetic variation for improved taxonomic resolution in phylogenetic studies [37–41]. The first two full approximately 15.2 kb *Junonia* mitochondrial genome sequences were reported from Old World species [40,41]. In 2016, 14 New World *Junonia* mitogenomes and four additional Old World mitogenomes were used to reconstruct patterns of diversification [10,42]. Subfamily Nymphalinae and genus *Junonia* formed monophyletic groups, but like previous findings using *COI* sequences, New World *Junonia* showed a lack of monophyly, consistent with earlier proposals of multiple *Junonia* invasions of the New World [10,18]. Separate invasions by different Old World species may have created each of the New World haplotype groups [5,10]. The haplotype groups A and B in *Junonia* were estimated to have diverged 2.31 ± 0.42 Ma, consistent with previous molecular clock estimates [3,19]. Divergence times for all other New World haplotypes were estimated: divergence of A_1_ and A_2_ 1.52 ± 0.31 Ma and the divergence of B_1_ from the rest of haplotype B 1.19 ± 0.29 Ma [10]. Peters & Marcus [5] recognized the distinctiveness of New World haplotype group C in high-elevation *J. vestina,* (more closely related to *J. lemonias* from Asia than it is to other New World haplotypes) and was estimated to have diverged 1.16 ± 0.32 Ma [5]. Consistent with previous studies, haplotypes A and B each formed monophyletic groups [10,16–19], but the placement of haplotype group C makes the mitochondria of the New World *Junonia* paraphyletic [5].

Recently, Cong *et al*. [23] analysed next-generation sequencing libraries from 11 New World *Junonia* species to describe two new species (*Junonia pacoma*; Pacific mangrove buckeye and *Junonia stemosa*; South Texas dark buckeye). Fixed diagnostic characters (morphological or molecular) that allow for the consistent separation of *J. stemosa* from the morphologically nearly identical *Junonia nigrosuffusa* could not be identified*,* so we will treat this form as a subspecies *J. nigrosuffusa stemosa* nov. stat. [23]. To date, the largest mitogenome-based *Junonia* phylogeny consists of 28 mitogenomes (15 *Junonia* species (eight New World, seven Old World), and one from each of the five other Junoniini genera) [26]. This analysis concluded that *Junonia* was monophyletic, haplotype groups A and B form monophyletic clades, but the New World *Junonia* are not monophyletic. The most likely sister clade to genus *Junonia* (though with weak bootstrap support) contains both African *Protogoniomorpha* and Asian *Yoma*, all consistent with a recent analysis of mitochondrial DNA barcodes from Old World *Junonia* species [25].

Many mitogenomes have now been published for *Junonia* (18 of 47 species) and other genera from tribe Junoniini (one species each from the five other genera) through GenBank (Dataverse electronic supplementary material, table S1). Here, we build upon this pre-existing knowledge by assembling additional full *Junonia* mitochondrial genomes (22 new species: 40 of the 47 described species), tribe Junoniini (16 additional species across five genera) and additional outgroup species into a phylogenetic analysis. To complement the mitogenomes, we conducted a phylogenetic analysis based on the nuclear ribosomal RNA (rRNA) repeat sequence (an 8–10 kb sequence containing three rRNA repeat subunits (2.8S, 18S and 28S), two internal transcribed spacers (ITS1 and ITS2), and 5′ and 3′ non-transcribed spacers) [43] from the same group of samples. Creating phylogenies using both types of sequences will aid in reconstructing patterns of reticulate evolution and describing biogeographic distributions for a rapidly diversifying clade.

## 2. Material and methods

### (a) Specimen collection, preparation and sequence generation

Ninety-seven specimens were analysed, consisting of 64 *Junonia* (40 of 47 described species), 21 additional specimens from tribe Junoniini and 11 outgroup species within subfamily Nymphalinae (Dataverse electronic supplementary material, table S1). We generated most data ourselves, and supplemented with additional sequences from GenBank. Specimens were identified based on morphology. DNA was extracted from a single leg per specimen using a Qiagen DNEasy Blood and Tissue Kit (Qiagen, Düsseldorf, Germany) either manually as previously described [18] or using the animal tissue DNA program on a Qiagen QiAcube. Samples were stored at −20°C before sequencing.

Sequence data were obtained using Ion Torrent (ThermoFisher Scientific, Waltham, Massachusetts, USA), Illumina MiSeq (San Diego, California, USA) or Illumina NovaSeq6000 sequencing (mean size of sequence libraries approximately 3.42 Gigabase pairs) [26]. Sequences were assembled and annotated to previously published reference sequences with Geneious 10.2.6 (Dataverse electronic supplementary material, table S1).

### (b) Mitogenome phylogeny

Phylogenetic reconstruction employed 97 mitogenome sequences from 64 *Junonia* specimens from 40 species, 21 specimens from other genera in tribe Junoniini and 12 outgroup specimens within subfamily Nymphalinae (Dataverse electronic supplementary material, table S1). Mitogenome sequences (Dataverse electronic supplementary material, file S1) were aligned in ClustalX 2.1 [44,45] and analysed using Bayesian inference with the GTR + I + G model (model selected by jModeltest 2.1.1 [46]) in MrBayes version 3.2.7 [47,48] for 10 million Markov chain Monte Carlo iterations, sampling every 1000 generations, with the first 25% of iterations discarded as burn-in. The trees produced were rendered in FigTree version 1.4.3 [49] and illustrated using Canvas X Draw.

### (c) Nuclear ribosomal RNA and internal transcribed spacer repeat phylogeny

Phylogenetic reconstruction employed 90 nuclear rRNA repeat sequences from 62 *Junonia* specimens, 19 specimens from other genera in tribe Junoniini and nine outgroup specimens from subfamily Nymphalinae (Dataverse electronic supplementary material, table S1). Our laboratory generated all of the nuclear rRNA repeat sequences, some of which were published previously [26–28,50–57], but are analysed in concert here for the first time, to our knowledge. When samples in the mitogenome dataset lacked GenBank Sequence Read Archives (SRAs) containing sufficient raw sequence data to assemble a nuclear rRNA repeat, they were excluded. Sequences were aligned (Dataverse electronic supplementary material, file S2) and analysed as described above.

## 3. Results

### (a) *Junonia* mitogenome phylogeny

**Figure 2.**
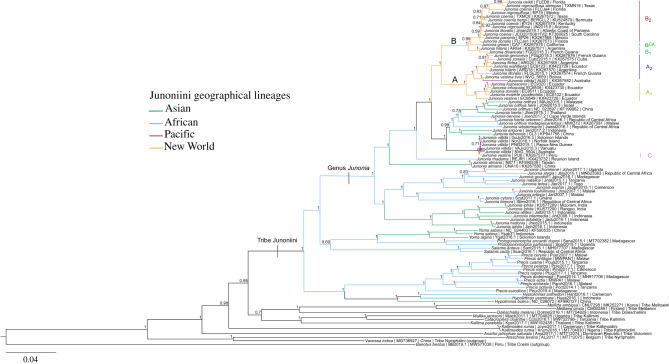
*Junonia* mitogenome Bayesian inference phylogeny (GTR + I + G model, best state likelihood = −170 867 and a deviation of split frequencies = 0.002109). Sixty-four *Junonia* mitogenomes, 21 other tribe Junoniini mitogenomes and 12 mitogenomes from other Nymphalinae tribes. The MrBayes Bayesian posterior probability values are given at each node. (Online version in colour.)

The mitogenome phylogeny was constructed using Bayesian inference with a GTR + I + G model with a best state likelihood of −170 867 and a final average deviation of split frequencies of 0.002109 (figure 2). Tribe Junoniini was monophyletic. The oldest node within Junoniini defines the divergence of *Precis* and *Hypolimnas* species from the rest of the tribe with a Bayesian posterior probability of 1 (figure 2). Sister to *Junonia* was a clade containing the genera *Protogoniomorpha*, *Yoma* and *Salamis*. *Salamis* diverges at the oldest node in the clade with low (0.69) Bayesian posterior probability, making the placement of this genus tentative. New World *Junonia* species resolve into a single large clade, with the exception of a single *J. villida* specimen from Australia grouping with New World sequences, and a single South American *J. vestina* specimen with Old World taxa. Individual New World species are not monophyletic in this analysis and interspecific relationships in the New World are unresolved (figure 2). Haplotype group A and subgroup A_2_ remain most prevalent in South American *Junonia* populations. Haplotype subgroup A_1_ originally thought to only occur in high-elevation *J. vestina* populations is shared by some *Junonia fuscescens*, *Junonia infuscata*, *Junonia zonalis* and *J. evarete* populations in Ecuador.

Haplotype group B is most prevalent in North and Central America, and Bermuda, and occurs with haplotype A in the Caribbean. Haplotype subgroup B_2_ is the most recently diverged within group B (figure 2) and is the predominant haplotype in the Western Hemisphere from Panama northwards. The B^CA^ haplotype group from *J. grisea* and other American Southwest *Junonia* is sister to the New World B_2_ subgroup. Subgroup B_1_ is the most divergent B haplotype lineage and only occurs in South American *Junonia* populations. The oldest nodes in both haplotype groups A and B show divergence of South American lineages (figure 2). Haplotype group C is restricted to *J. vestina* in Peru and is unique in that it does not cluster with other New World haplotype groups but instead with a *J. villida* mitogenome clade (Old World species with an Indo-Pacific distribution). Similarly, a single *J. villida* mitogenome is sister to the New World haplotype A_2_ clade, suggesting that there may be recent or historical gene flow between populations of New World *J. vestina* and Indo-Pacific *J. villida* populations.

In contrast with the New World, prior studies concluded that individual Old World *Junonia* species were monophyletic based on limited sampling of species and populations. The current analysis of complete mitochondrial genomes suggests a lack of monophyly in at least some Old World *Junonia* species (figure 2). For example, *J. orithya* and *Junonia hierta* form two separate lineages, one in Asia and one in Africa, which are more closely related to the sympatric lineage of the other species than they are to conspecific allopatric lineages. This suggests that there has either been remarkable parallel morphological and colour pattern evolution in these lineages, or that there may be lateral transfer and introgression of mitochondrial haplotypes between them. Another species pair that may be experiencing introgression and ongoing geneflow are *Junonia iphita* and *Junonia hedonia*. The *J. iphita* mitogenome sequence from Indonesia forms a clade with sympatric sequences from *J. hedonia* rather than with *J. iphita* sequences from elsewhere in Asia. This suggests that lateral transfer events and introgression is more widespread in Old World *Junonia* than previously appreciated.

### (b) *Junonia* nuclear ribosomal RNA repeat phylogeny

**Figure 3.**
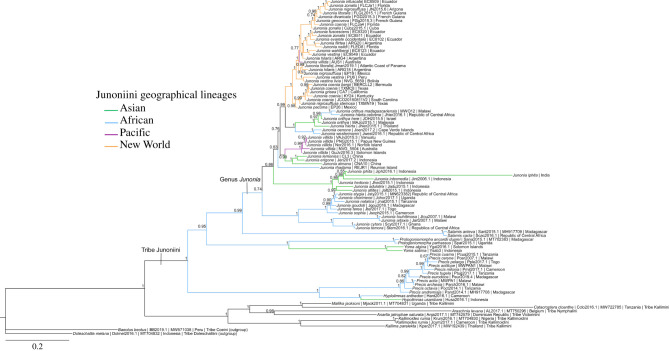
*Junonia* complete rRNA repeat Bayesian inference phylogeny (GTR + I + G model, best state likelihood = −119 000 and an average deviation of split frequencies = 0.003566) based on 62 *Junonia* specimens, 19 other sequences from tribe Junoniini and nine outgroup species from tribes within subfamily Nymphalinae. The Bayesian posterior probability values determined by MrBayes are given at each node. (Online version in colour.)

The nuclear rRNA repeat phylogeny was constructed using Bayesian inference with a GTR + I + G model with a best state likelihood of −119 000 and a final average deviation of split frequencies of 0.003566 (figure 3). Tribe Junoniini and its component genera are monophyletic. Nearly all of the New World *Junonia* nuclear rRNA repeats form two distinct clades. One clade is restricted to North America and Bermuda. The other clade contains specimens from South, Central, and parts of North America, and the Caribbean. Some New World *Junonia* species include individuals assigned to both clades. As a whole, the New World *Junonia* are monophyletic with only a single exception: the same Australian *J. villida* specimen that grouped with New World mitogenomes also clusters with New World nuclear rRNA repeats. The *J. vestina* specimen with a distinct mitogenome (haplotype group C) that had grouped with the Old World *J. villida* clade in the previous analysis (figure 2) is the sister taxon to another South American *J. vestina* sample within the New World clade in the nuclear rRNA repeat phylogeny (figure 3).

The nuclear rRNA repeat phylogeny for Old World *Junonia* taxa is consistent with the mitogenome phylogeny with two major differences. The *J. villida* clade, for which a *J. vestina* sequence from the New World was a sister clade in the mitogenome tree, is more closely associated with a group of Asian *Junonia* species (*J. lemonias*, *Junonia erigone* and *Junonia almana*) in the nuclear rRNA repeat phylogeny. The second is the sister clade to this Asian clade in the mitogenome tree, is the sister clade to the New World *Junonia* in the rRNA repeat phylogeny (figure 3). This clade, which includes both African and Asian lineages, contains *J. orithya* and *J. hierta.* The African lineages of *J. orithya* and *J. hierta* form a clade together and Asian lineages from these two species and are interspersed with one another, similar to the results of the mitogenome analysis. By contrast, the nuclear rRNA repeats from *J. iphita* form a monophyletic group and do not show the affinity with *J. hedonia* sequences as observed using mitogenomes (figure 2).

The relationships within tribe Junoniini based on rRNA repeats are consistent with the mitogenome analysis except for the placement of *Salamis*, which is placed as sister taxon to *Junonia* with a very high probability value (0.99). The relationships of the outgroup species are consistent with the mitogenome analysis, with the exception of tribe Kallimini, which is paraphyletic in the nuclear rRNA repeat analysis. *Mallika jacksoni* and *Catacroptera cloanthe* are assigned to tribe Kallimini as expected, but *Kallima paralekta* clusters with tribe Kallimoidini within subfamily Nymphalinae (figure 3).

## 4. Discussion

### (a) Tribe Junoniini, the sister clade of *Junonia* and the paraphyly of Kallimini

The clade defined by the oldest node within the Junoniini (figures 2 and 3) contains the monophyletic genera *Precis* and *Hypolimnas,* as reported previously [3,24,25]. The sister clade to *Junonia* differs between mitochondrial and nuclear analyses. The mitogenome sister clade contains the genera *Protogoniomorpha*, *Yoma* and *Salamis*, with *Salamis* as the outgroup to the other two genera, consistent with some prior phylogenetic analyses [3,24–26]. Pyrcz *et al*. [25] found genus *Salamis* embedded within *Junonia*. Our rRNA repeat phylogeny shows *Salamis* as sister to genus *Junonia* (posterior probability value of 0.99), with the next further outgroup containing the genera *Yoma* and *Protogoniomorpha*.

*Kallima* (tribe Kallimini) originally served as a catch-all genus for Asian and African nymphalid butterflies masquerading as leaf mimics [58]. Based on genitalia and behavioural characteristics, it was later determined that genus *Kallima* is restricted to Asia, and African leaf mimics and were reassigned to three other Nymphalinae genera (*Junonia* (Junoniini)*, Mallika* (Kallimini) or *Kallimoides* (Kallimoidini)) [29,59,60]. Tribe Kallimini comprised the genera *Kallima*, *Mallika* and *Catacroptera* and was viewed as the sister clade to the Junoniini, since most Kallimini and the basal character state within Junoniini is leaf mimicry [59]. Molecular phylogenetics has consistently placed tribe Melitaeini as the sister to the Junoniini, with tribe Kallimini placed as a farther outgroup [29,61]. While the mitogenome tree (figure 2) is consistent with these results, phylogenetic reconstruction using the rRNA repeat region (figure 3) shows genera *Mallika* and *Catacroptera* (both African genera) forming a monophyletic clade. Genus *Kallima* falls outside this grouping and as sister to *Kallimoides*, with a Bayesian probability value of 1 (figure 3). These differences could be attributable to limited taxon sampling among outgroups within the rRNA repeat phylogeny (figure 3). Both the Junoniini and Kallimini show close molecular phylogenetic associations with the Melitaeini in our mitogenome reconstruction (figure 2) and in previous studies [29,62,63], but nuclear rRNA repeat sequences, helpful for inferring higher level taxonomic relationships within the Nymphalinae, are not yet available from the Melitaeini.

### (b) New World *Junonia*

Species-level relationships in the New World *Junonia* were unresolved by mitogenomes (figure 2), consistent with earlier DNA barcode and mitogenome studies [3,5,16–19,23]. Many New World species include individuals that carry both A and B haplotype groups. Similarly, most New World *Junonia* species do not form monophyletic groups based on nuclear rRNA repeats. This is indicative of gene flow between New World *Junonia* species and is consistent with prior observations of hybridization between many of these species [20,23,30,32]. Based on the mitogenome phylogeny (figure 2), the New World *Junonia* is monophyletic with two exceptions. The first is a *J. vestina* sample that possesses haplotype group C and is most closely related to a lineage of *J. villida* from the Indo-Pacific. This finding differs from that of Peters & Marcus [5], which placed *J. vestina* as the sister taxon to *J. lemonias*. Peters & Marcus [5] only included single representatives of most *Junonia* species, including *J. villida,* so this discrepancy can be attributed to limited sampling*.* In both the current study and Peters & Marcus [5], a single *J. villida* sample from Australia is most closely related to the haplotype group A_2_ in the New World. These findings signify that long-range dispersal and gene flow across the Pacific may be occurring and is of importance to understanding the relationships between the Old World and New World species, as well as the origins of the New World *Junonia*.

Complete nuclear rRNA repeats occur at high copy number in the genome and are easily recovered from the same genome skimming datasets used for assembling whole mitogenomes (figure 3; [43]). Phylogenetic analysis of *Junonia* nuclear rRNA repeats did not recover the same clades found through analyses of mitogenomes, nor do rRNA repeats resolve New World species-level relationships. Like the mitogenome, there is an apparent geographical signal, with most North American specimens forming one rRNA repeat lineage, while a second is made up of Central and South American, Caribbean and a few specimens from southern portions of North America. Based on nuclear rRNA repeat sequences, the New World *Junonia* are monophyletic, except for an Australian *J. villida* sample that also groups with the New World in the mitogenome phylogeny. Unlike the mitogenome phylogeny, the nuclear rRNA repeats of all *J. vestina* samples fall within the New World rRNA clade. This further supports the hypothesis that gene flow between New World *J. vestina* and the Indo-Pacific *J. villida* by means of long-distance dispersal across the Pacific, followed by hybridization, may be ongoing since individual specimens do not share the same level of molecular affinity with one another, with some forming clades with the other taxon.

### (c) Old World *Junonia*

Previous phylogenetic studies of Old World *Junonia* could not test for monophyly or make strong statements about species-level relationships, because only a single sample of each species was used [9,16–18,24–26,29]. Kodandaramaiah & Wahlberg [3] was exceptional in including multiple specimens from some Old World species comparable with this analysis. First, specimens from Asian and African populations of *J. hierta* and *J. orithya* form clades based on geography rather than taxonomic species assignment here (figures 2 and 3) and in Kodandaramaiah & Wahlberg [3], though they did not comment on the pattern in the text of their paper. Also consistent with Kodandaramaiah & Wahlberg [3] is the placement of this clade as sister to the New World *Junonia*, although our mitogenome data includes *J. villida* within the clade. Further, mitogenomes from *J. iphita* pair with either *J. hedonia* or *Junonia atlites* (figure 2). By contrast, in the analysis of rRNA nuclear repeats (figure 3), the *J. iphita* sequences form a monophyletic clade and none are sister to *J. hedonia*. A recent lateral transfer of the *J. hedonia* mitogenome to *J. iphita* in Indonesia seems likely as the sequences are nearly identical, but additional sampling and sequencing from these and other species in this lineage would clarify patterns of organelle capture*.* What seems increasingly clear is that while lateral transfer events may be most frequent in the New World *Junonia*, it also occurs in some Old World lineages, contributing to reticulate evolution of the genus as a whole.

**Figure 4.**
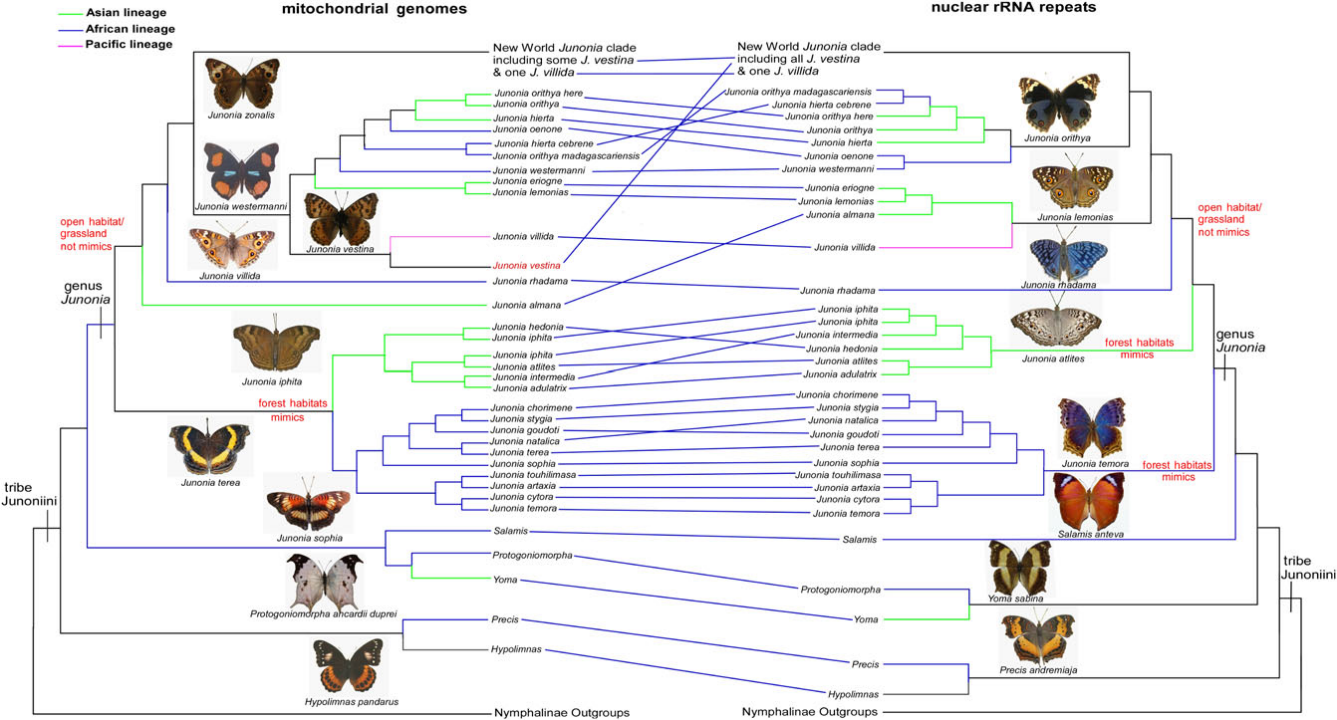
Modified molecular phylogeny of the Old World *Junonia*. Interpretations based on mitogenome and rRNA repeat phylogenetic reconstructions from figures 2 and 3. Geographical origins, habitat preferences and the display of mimicry of lineages are indicated. (Online version in colour.)

Despite instances of apparent lateral transfer, the Old World *Junonia* form distinct molecular clades made up of species that share phenotypic features (figure 4). Although there are small differences between the two different phylogenetic analyses in respect to species placement, all Old World species can be grouped based on geography, habitat type and mimicry strategy. Some clades defined by the oldest *Junonia* nodes in the mitogenome phylogeny (figure 4) consist of two lineages of forest-dwelling species. The first is an Asian forest-dwelling butterfly lineage containing species (*J. iphita*, *J. atlites*, *Junonia intermedia*, *Junonia adulatrix* and *J. hedonia*) that at rest have closed wings that masquerade imprecisely as leaves [58]. The second lineage includes African species that are also forest-dwelling but most are considered to be very good leaf mimics [24] (figure 4). There is one exception, *Junonia sophia,* which is thought to be a Batesian mimic of the false diadem butterfly (*Pseudacraea lucretia*) [59]. Within this African *Junonia* lineage, there are two subclades that cluster based on similar coloration of the dorsal wing surfaces (either blue or brown).

The next large *Junonia* clade in the mitogenome phylogeny includes all New World *Junonia* species as well as some Asian (*J. orithya, J. hierta, J. lemonias, J. villida, J. erigone and J. almana*) and African species (*J. orithya, J. hierta, Junonia oenone, J. westermanni and Junonia rhadama*). These are grassland or open habitat specialists, and none are considered to be mimics (figure 4, [59,64]). Consistent with prior studies, this lineage originated in Africa, dispersed to Asia, and then returned to Africa, perhaps several times [3]. Later, this lineage established the New World *Junonia* radiation. The rRNA repeat phylogeny yields similar results to the mitogenome analysis, with phylogenetic trends based on habitat type, mimicry and geography, but differences do exist. The clade defined by the oldest node within *Junonia* recovered by this analysis supports an African origin for this genus, as all species in this clade are forest-dwelling and restricted to Africa (figure 4), reinforcing mitogenome results and an earlier three-gene analysis [3]. Like the mitogenome phylogeny, all species within this clade are mimics. The next clade consists of Asian and Indo-Pacific *Junonia*, with one exception, *J. rhadama* that occupies Madagascar and several other Indian Ocean islands. The first major split in this clade is by habitat type with forest-dwelling imperfect leaf mimics and grassland/open habitat lineages. Forest-dwelling species include the *J. iphita* and *J. hedonia* complex discussed above in the mitogenome phylogeny and the same pattern is observed here. Open habitat species include *J. rhadama*. The remaining open habitat species are more similar morphologically to New World taxa than they are to other Old World species. A key difference in the composition of this clade exists between our phylogenetic analyses. In the mitogenome analysis, *J. villida* is the earliest diverging species and sister to the New World *Junonia* (figure 4), while in the nuclear rRNA repeat analysis, the sister clade is limited to African *J. westermanni,* and *J. oenone* and African/Asian *J. orithya* and *J. hierta*, and the remaining taxa (Asian: *J. villida, J. lemonias*, *J. erigone* and *J. almana*) are transferred into a separate clade (figure 4). This difference between the mitogenomic and nuclear rRNA repeat phylogenies raises the intriguing possibility that the New World *Junonia* were established with contributions from two Old World *Junonia* lineages: one containing *J. villida* which crossed the Pacific Ocean and a second containing *J. orithya* and *J. hierta*, which could have crossed either the Atlantic or Pacific Ocean to reach the New World. Thus, several of the early hypotheses for the origin of the New World *Junonia* may not have been mutually exclusive after all and instead may turn out to be simultaneously correct [12,13,15].

## 5. Conclusion

Compared with many other butterfly taxa, *Junonia* have particularly effective dispersal abilities, allowing them to colonize remote new habitats, perhaps in some cases repeatedly. This creates scenarios where resident and new immigrant *Junonia* are sympatric and lateral transfer is possible through hybridization and reticulate evolution. This appears to have taken place both in the Old World and New World. The large number of New World *Junonia* (18 species) that have evolved in a very short period of time (2–4 Myr) suggest that speciation in this group has been accelerated compared to the older lineages in the Old World (28 species) which have diverged since the origins of the genus 15–27 Ma [3]. The variety of new ecological niches (especially larval host plant associations) presented by new habitats, in combination with frequent lateral transfer of adaptive genes and traits, and reticulate evolution may have contributed to the substantially greater rate of speciation in the New World, making this system similar to the explosive species radiation events such as the Lake Victoria cichlids [65] or the Hawaiian *Drosophila* [66].

To address the challenges of species delimitation in *Junonia* in the face of reticulate evolution*,* one recent study successfully employed complete Z chromosome sequences [23]. Although their dataset only included specimens from some New World *Junonia* taxa, they were able to resolve the species into monophyletic clades. Accumulating complete Z chromosome sequences from the rest of the genus will provide an interesting comparison to results from analyses of mitochondrial genomes and nuclear rRNA repeats, and together this may permit better delimitation of the remaining species, further resolve species-level phylogenetic relationships, and should be considered for future phylogenetic studies of this genus.

## Data Availability

All sequences and SRAs have been made submitted to be publically available through GenBank and accession numbers for all data used in the analyses can be found in the Dataverse electronic supplementary material, table S1 (https://doi.org/10.34990/FK2/O9UKCE/LQDEC8) which has been deposited through the online data repository Dataverse. Sequence alignment data is available as electronic supplementary files through the online data repository Dataverse for both the full mitochondrial genomes (https://doi.org/10.34990/FK2/O9UKCE/5S8ZJ8) and rRNA repeats (https://doi.org/10.34990/FK2/O9UKCE/YYM2ZT).
